# Identifying potential co-expressed genes and molecular mechanisms linking post-COVID-19 and Guillain-Barre syndrome through neutrophil extracellular trap-related genes

**DOI:** 10.3389/fneur.2025.1447725

**Published:** 2025-05-13

**Authors:** Jie-Hua Su, Dan-Yu Lin, Xiao-Huan Liu, Jie-Li Zhang, Zhong-Gui Li, En-Xiang Tao, Kai-Xun Huang

**Affiliations:** ^1^Department of Neurology, The Eighth Affiliated Hospital, Sun Yat-sen University, Shenzhen, China; ^2^Department of Neurology, Sun Yat-sen Memorial Hospital, Sun Yat-sen University, Guangzhou, China

**Keywords:** Guillain-Barré syndrome, post-COVID-19, SARS-CoV-2, computational biology, bioinformatics, inflammation, neutrophil extracellular traps

## Abstract

**Introduction:**

Neutrophil extracellular traps (NETs) play a pivotal role in immunity and autoinflammatory disease, leading us to hypothesize that NETs are crucial in Guillain-Barre Syndrome (GBS) after SARS-CoV-2 infection.

**Methods:**

By collecting six Gene Expression Omnibus (GEO) datasets from the GEO database and dividing them into discovery and validation sets, we screened differentially expressed genes (DEGs) within the discovery set, with further analyses using functional enrichment analysis. Using single-sample gene set enrichment analysis (ssGSEA), we assessed immune cell infiltration in both coronavirus disease 2019 (COVID-19) and GBS datasets. NETs-related genes (NETRGs) were identified through a protein–protein interaction (PPI) network and NETs gene datasets. Finally, candidate drugs were screened using Connectivity Map.

**Results:**

In this study, a total of 3254 DEGs were identified from the COVID-19 dataset, and 692 DEGs were obtained from the GBS dataset. Among these, 145 co-expressed DEGs were obtained. Bioinformatics functional analysis indicated that co-expressed DEGs were predominantly gathered in immune-related and inflammatory response pathways. Employing various algorithms, we identified MMP9, CAMP, and CASP1 as NETRGs, demonstrating good discriminatory capacity in COVID-19 and GBS. Notably, neutrophils and macrophages were identified as co-upregulated differential immune infiltrating cells significantly associated with both COVID-19 and GBS. Moreover, we identified 10 candidate drugs for patients with post-COVID-19 GBS.

**Conclusion:**

In conclusion, MMP9, CASP1, and CAMP were identified as promising biomarkers and potential targets for therapy of post-COVID-19 GBS.

## 1 Introduction

The coronavirus disease 2019 (COVID-19) pandemic, caused by the severe acute respiratory syndrome coronavirus 2 (SARS-CoV-2) virus, has had a significant influence on the global public health scene. Alongside its respiratory symptoms, COVID-19 has been linked to various neurological complications (1–5), such as myositis, stroke, encephalitis, acute meningitis, and Guillain-Barré syndrome (GBS). Existing research predominantly focused on the central nervous system consequences of COVID-19, such as cognitive dysfunction, while peripheral nervous system damage, including GBS, and its underlying mechanisms have received less attention. However, GBS is widely recognized as the leading cause of acute paralysis, often emerging in the aftermath of infections, particularly viral infections (6). The significant complications of GBS, such as autonomic dysfunction and respiratory failure, underscore the critical importance of post-COVID-19 GBS. Instances of GBS cases associated with COVID-19 have been reported in at least 23 countries (7), demonstrating a prevalence of 15 cases per 100,000 population years through meta-analysis (8). An Italian study (9) found increased GBS in COVID-19 patients, indicating a higher risk within the infected population. Notably, even among non-hospitalized COVID-19 patients, an increased risk of GBS (10) has been documented. Furthermore, post-COVID-19 GBS has been recognized as part of the “long-term COVID-19 syndrome,” exhibiting more unfavorable outcomes compared to non-COVID-19 GBS (10, 11). This suggests a persistent delay in symptoms and poor clinical outcomes for individuals with post-COVID-19 GBS.

GBS has classically been considered as a demyelinating disease caused by phagocytosis of myelin by macrophages (12), but the axonal form is now widely recognized as another major subtype (13). Recent studies suggest acute inflammatory demyelinating polyneuropathy (AIDP) may be more prevalent in COVID-19-associated cases (14), though evidence remains inconclusive with diverse subtype presentations reported in ongoing case series. This observation suggests a potential link between the immune response to COVID-19 and the pathogenesis of AIDP, possibly due to cross-reactive immune responses or molecular mimicry between SARS-CoV-2 antigens and peripheral nerve components. Unlike directly viral-induced pathogenesis, post-COVID-19 GBS predominantly arises from immune dysregulation, including molecular mimicry, cytokine storms, and autoimmunity. These mechanisms often act synergistically, suggesting a complex interplay between host genetics and pathogen-induced inflammation (15). In fact, it is reported that post-COVID-19 GBS was an immune mediated disease rather than direct SARS-CoV-2-mediated cause (8), indicating a significant role of immune dysregulation and inflammation in its pathogenesis. SARS-CoV-2 infection activated the adaptive immune system, prompting interactions between T-cells and B-cells that induce antibody production, which can target nerve and myelin cells. Neutrophil extracellular traps (NETs) serve as a defense mechanism against infection, primarily targeting large pathogens, but they are also implicated in the pathogenesis of an expanding number of immune-mediated disorders (16). A study by Prével et al. (17) revealed a significant correlation between circulating markers of NETs and survival rate in COVID-19 patients, highlighting the potential for targeting neutrophil extracellular trap formation as a preventive measure against disease exacerbation. The high elevation of neutrophil proportion was observed among GBS patients (18), while the recruitment cascade and highly activation of neutrophil may trigger NETs formation. Indeed, inflammation-induced NETs formation can exacerbate inflammation and contribute to auto-antibody production (19). Emerging studies have indicated that NETs may be promising therapeutic targets for immune-mediated disorders, but their role in post-COVID-19 GBS pathogenesis is still unclear. Therefore, further comprehensive research is needed to elucidate the function of NETs in post-COVID-19 GBS and to explore their potential as therapeutic targets.

Notably, only a limited number of studies have investigated the underlying mechanisms of COVID-19-related GBS using bioinformatics analysis (20, 21), with particular emphasis on the role of NETs. In this study, we conducted a transcriptomics-based bioinformatics analysis to uncover alterations in gene expression associated with COVID-19 and GBS co-occurrence related to NETs genes, which may illuminate the pathogenic mechanisms of post-COVID-19 GBS.

## 2 Materials and methods

### 2.1 Datasets acquisition and processing

The datasets GSE31014, GSE213313, GSE215865, GSE195796, GSE191088, and GSE200274 were collected from the Gene Expression Omnibus (GEO) database.^1^ For identifying common hub genes, R software (version V4.2.1) was employed, with GSE31014 and GSE213313 selected as the discovery sets. GSE215865 was used for the hub genes validation. Furthermore, GSE195796 (datasets of COVID-19) were analyzed to explore the associations between hub genes and clinical characteristics, while GSE191088 and GSE200274 were utilized to investigate the relationship between hub genes and COVID-19 vaccination. Raw data or series matrix files underwent background correction and normalization before subsequent analyses. All data in this study were obtained from publicly available databases and adhered to ethical standards. Differentially expressed genes (DEGs) were identified using the limma package in R, applying cutoff criteria of |logFC (fold change)| > 1.5 and *p* < 0.05. Co-expressed DEGs in both COVID-19 (GSE213313) and GBS (GSE31014) datasets were determined through intersection.

### 2.2 Enrichment analyses of DEGs

The Gene Ontology (GO) and Kyoto Encyclopedia of Genes and Genomes (KEGG) enrichment analyses, encompassing biological processes (BP), cellular components (CC), and molecular functions (MF), serve as a fundamental tools for exploring functional enrichment in medical research. In order to gain insights into the underlying disease mechanisms, GO/KEGG enrichment analysis of the overlapping genes was conducted using the “clusterProfiler” package in R. This analysis aimed to elucidate potential biological pathways and functions associated with the disease. The statistical significance for GO/KEGG enrichment analysis was set at adjusted *p* < 0.01.

### 2.3 PPI network construction and hub gene analysis

To investigate the potential interaction relationships among the DEGs, the Search Tool for Retrieval of Interacting Genes (STRING) database (version 11.5; www.string-db.org) (22) was utilized to construct a protein–protein interaction (PPI) network. A minimum required interaction score of 0.400 was applied, and non-interacting genes were omitted from the network visualization. Hub gene identification involved topological analysis using five different algorithms (MNC, MCC, EPC, Degree, and Closeness) with the CytoHubba plugin in Cytoscape software. Notably, network feature measurements of nodes were used to determine their significance in biological networks and identify central elements within these networks. Visualizations of the top 10 DEGs from each algorithm were obtained, and NETRGs were determined by the intersection of all five algorithms and the neutrophil extracellular trap-related genes list from a previous study (23).

### 2.4 Immune infiltration analysis

To assess immune infiltration between control and specific (GBS or COVID-19) samples, we employed single-sample gene set enrichment analysis (GSA). This approach quantifies the extent of gene set enrichment in each sample within a given dataset. By using the GSVA package in R (24), we calculated normalized enrichment scores for each immune category. A heat map portraying immune infiltration in the samples was generated using the “pheatmap” package. The “ggplot2” package was used to visualize the levels of immune cells and immune function between control and specific (GBS or COVID-19) samples. Moreover, the correlation between hub genes and immune infiltration in specific (GBS or COVID-19) samples was assessed using the “ggplot2” package “pheatmap” package. Correlation analysis was assessed using Pearson correlation, with statistical significance accepted at *p* < 0.05.

### 2.5 Validation of hub genes expression

The mRNA expression of the identified NETRGs underwent validation within GSE215865, encompassing 329 samples from COVID-19 patients and 65 samples from normal controls (limited to subjects with non-repetitive and blood sample collection at admission). Unfortunately, the validation process for the GBS dataset was discontinued due to insufficient clinical transcriptomic samples. Furthermore, the GSE213313 datasets provided gene expression data at multiple time points during COVID-19 infection. As a result, GSE195796 was used to evaluate the temporal heterogeneity of NETRGs in COVID-19. Additionally, GSE191088 and GSE200274 datasets were explored to investigate the relationship between NETRGs and COVID-19 vaccination. Visual representation of NETRGs comparison across these diverse scenarios was facilitated through the ggplot2 package. Using the R packages “glmnet” and “pROC,” the receiver operating characteristic (ROC) curve for NETRGs was generated to evaluate their diagnostic capability in identifying disease onset for both COVID-19 and GBS. In the aforementioned scenarios, statistical analysis employed the *T*-test or the Wilcoxon test for two-group comparisons, and the Kruskal-Wallis test or ANOVA test for comparing three or more groups, depending on data normality.

### 2.6 Identifying drug candidates

The cMap database^2^ (25), containing gene expression profiles from treating 54 cell lines with 5,000 small-molecule compounds, emerged as a pivotal resource. It has primarily been used for finding new repurposed drugs for primary disease treatment, rather than addressing drug resistance in existing treatments. The PPI network-derived DEGs underwent screening in cMap, unveiling potential therapeutic compounds. Negatively related drugs with an enrichment score of <0 were considered effective compounds for the treatment of COVID-19 infection combined with GBS.

## 3 Results

### 3.1 Dataset information and identification of DEGs

**Figure 1:**
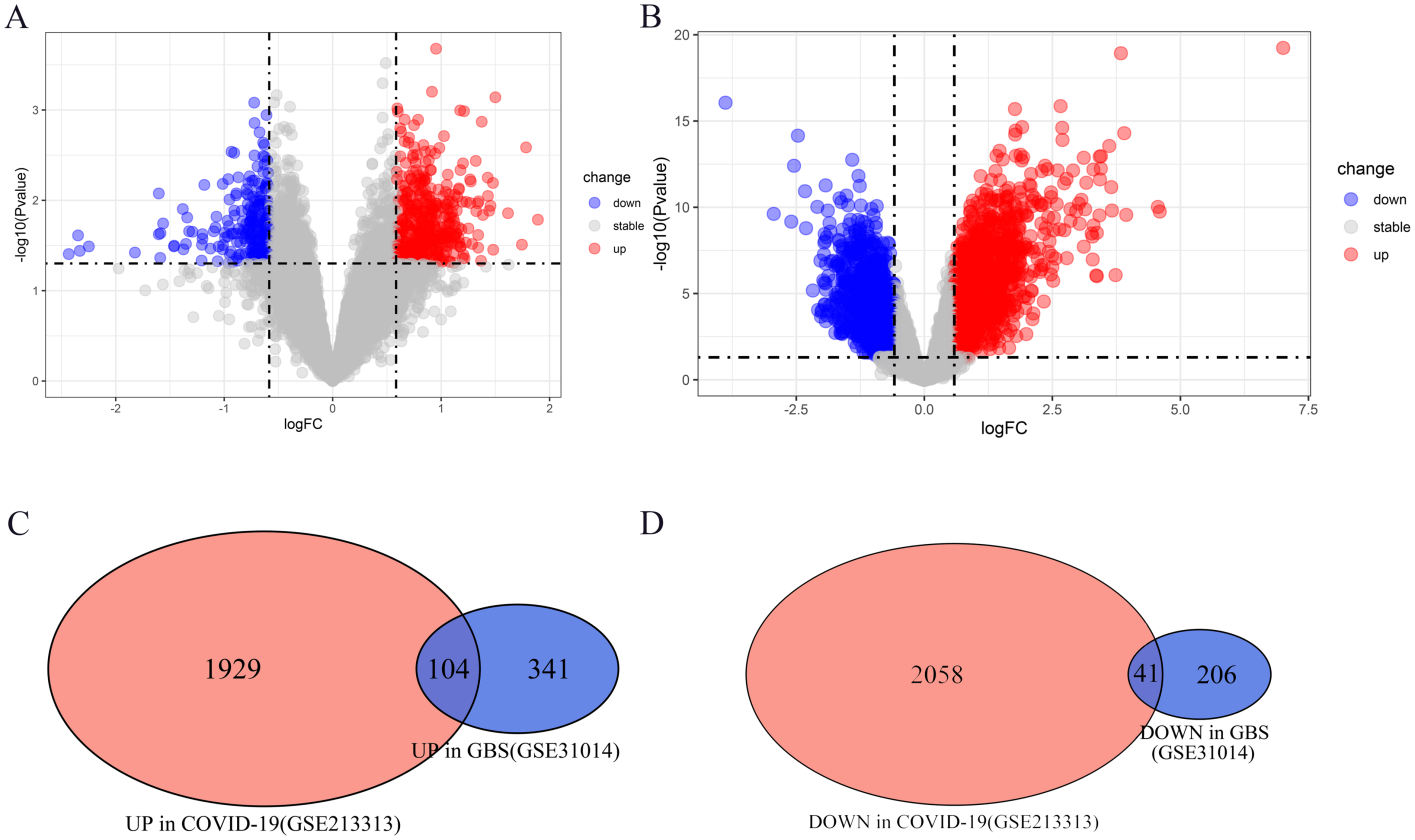
The co-expressed differentially expressed genes (DEGs) between GBS and COVID-19. **(A,B)** The volcano plots show the distribution of DEGs, with a large number of genes significantly upregulated or downregulated in GSE31014 (GBS) and GSE213313 (COVID-19). **(C,D)** The Venn diagrams reveal 145 co-expressed DEGs (104 co-upregulated and 41 co-downregulated) shared between GBS and COVID-19, indicating potential common biological processes involved in both diseases.

Table 1 provides comprehensive information about the selected datasets. Compared with normal samples, a total of 692 DEGs (445 upregulated and 247 downregulated genes) in GSE31014, and 3,254 DEGs (1,578 upregulated and 1,676 downregulated genes) in GSE213313 were identified in GBS and COVID-19 samples. The volcano plots in Figures 1A,B display all DEGs, while the Venn diagram in Figures 1C,D illustrates 145 co-expressed DEGs (104 co-upregulated and 41 co-downregulated genes) within the discovery datasets of GBS and COVID-19.

**Table 1:** Basic information of GEO datasets used in the study.

GEO Series	Data Type	Platform	Sample description and size (n)	Tissue
GSE31014	Discovery set	GPL96	GBS patient(7) normal control(7)	Peripheral blood
GSE213313	Discovery set	GPL21185	Time 1: Healthy control (11); Non-critical COVID-19 infection patients(15); Critical COVID-19 infection patients (19) Time 2: Non-critical COVID-19 infection patients (11); Critical COVID-19 infection patients (18) Time 3: Non-critical COVID-19 infection patients (7); Critical COVID-19 infection patients (13)	Peripheral blood
GSE215865	Validation set	GPL24676	Individuals with COVID-19 infection (329); healthy controls (65)	Peripheral blood
GSE195796	Validation set	GPL24676	Acute COVID-19 infection patients (31); Post-acute COVID-19 infection patients (33)	Peripheral blood
GSE191088	Validation set	GPL24676	Individuals before inactivated SARS-CoV-2 vaccine (18); Individuals after inactivated SARS-CoV-2 vaccine (18);	Peripheral blood
GSE200274	Validation set	GPL24676	Individuals with SARS-CoV-2 mRNA vaccination (6); Individuals without SARS-CoV-2 mRNA vaccination (6)	Peripheral blood

GEO, Gene Expression Omnibus; GSE, Gene Expression Omnibus Series; GPL, Gene Expression Omnibus Platform; COVID-19, Coronavirus disease 2019; SARS-CoV-2, Severe acute respiratory syndrome coronavirus 2; GBS, Guillain-Barré syndrome.

### 3.2 GO and KEGG enrichment analysis

**Figure 2:**
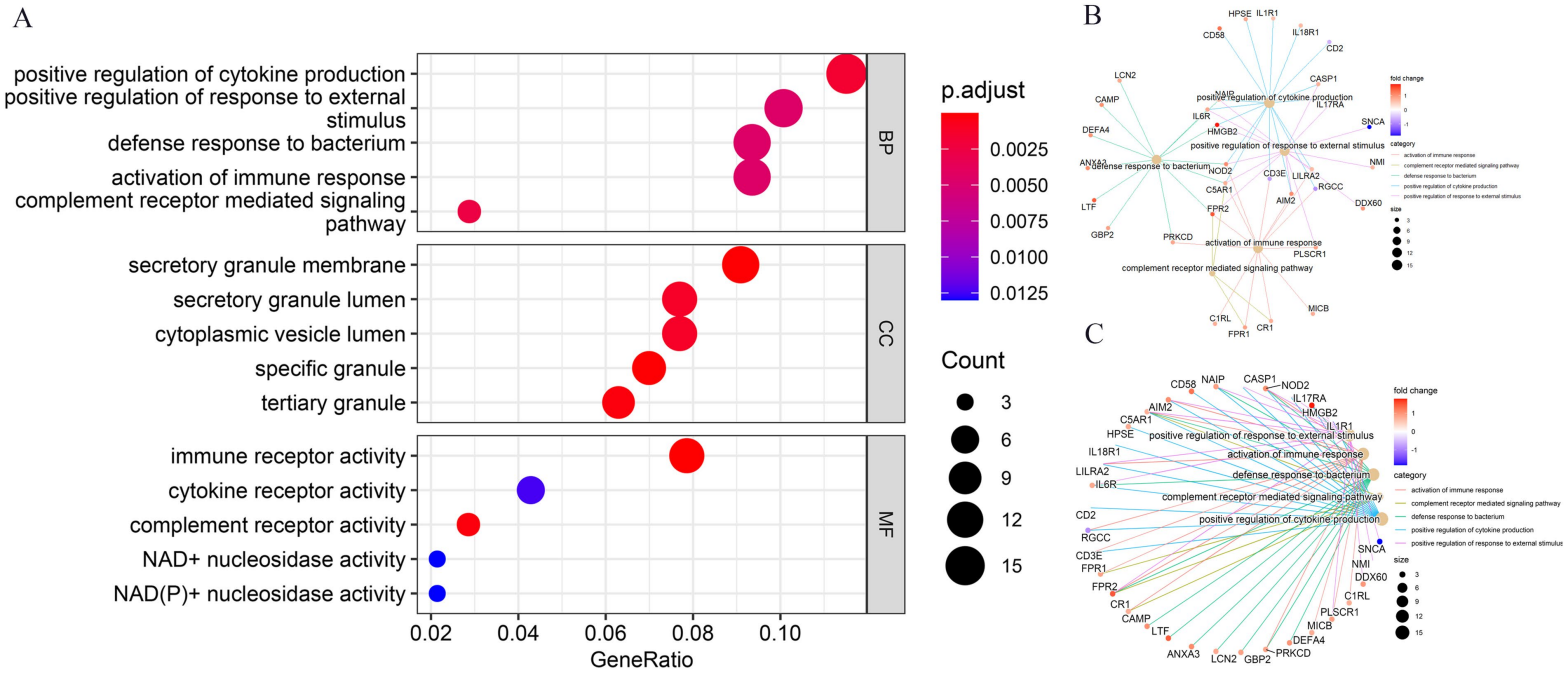
Gene ontology (GO) enrichment analysis of co-expressed DEGs. This figure illustrates the results of GO enrichment analysis for the co-expressed DEGs. The bubble diagram **(A)** highlights the top 5 enriched GO terms (ranked by adjust *p*-value shown by bubble diagram), primarily related to immune responses and inflammatory processes. The horizontal axis represents the gene count, and the vertical axis represents the GO terminology. The size of the dots represents the number of enriched genes, and the shades of color represent adjust *p*-value. **(B)** GO-BP enrichment analysis **(C)** GO enrichment analysis shown by circle diagram.

**Figure 3:**
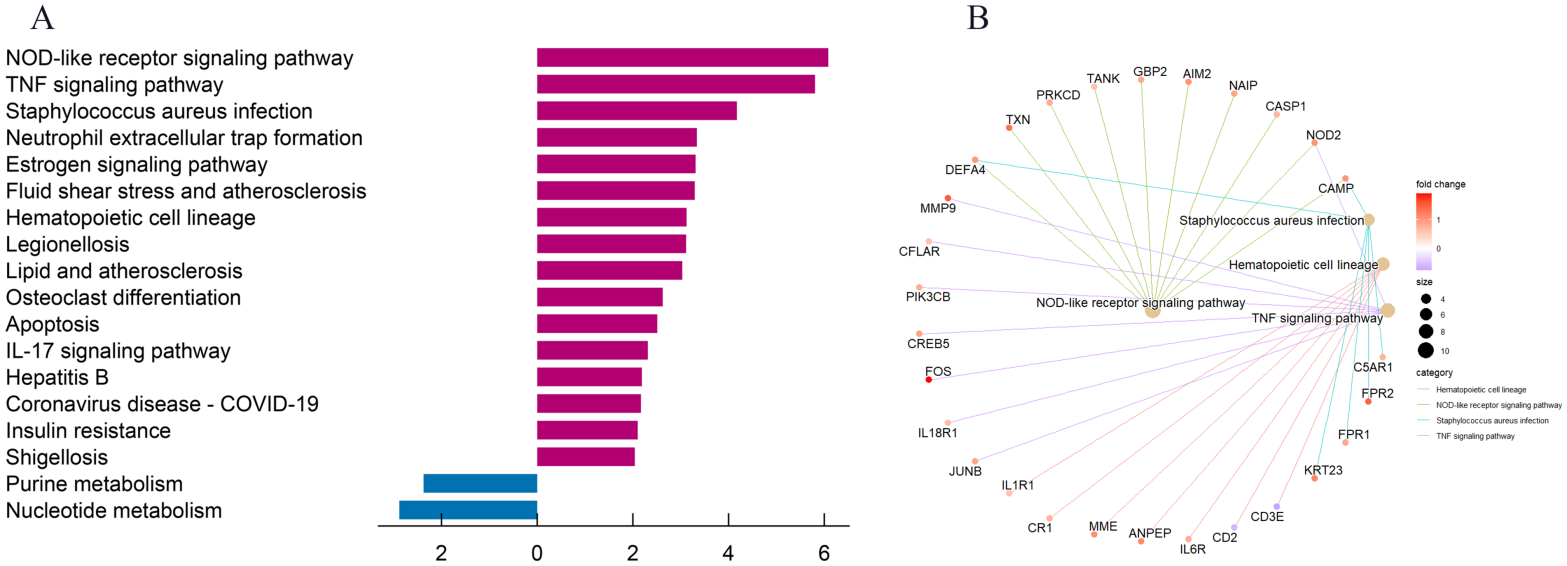
Kyoto Encyclopedia of Genes and Genomes (KEGG) pathway enrichment analysis of co-expressed DEGs. **(A)** The column diagram depicts the enriched KEGG pathways of co-expressed DEGs, ranked by their adjusted *p*-values. The horizontal axis represents the gene count, with red columns representing unregulated pathways and blue columns representing downregulated pathways. The column diagram shows the most significantly enriched pathways, including those related to neutrophil extracellular trap formation, TNF signaling, and other immune-related pathways. **(B)** KEGG enrichment analysis shown by circle diagram. The circle diagram offers a broader perspective of all enriched pathways, emphasizing the central role of immune and inflammatory mechanisms in both GBS and COVID-19.

The GO analysis of the aforementioned 145 co-expressed DEGs identified enrichment in biological processes linked to enhancing immune responses, such as cytokine production, complement receptor signaling, defense against bacteria, activation of an immune response, positive regulation of response to external stimuli, and regulation of inflammatory response. With respect to cellular components, DEGs co-expressed between GBS and COVID-19 patients were primarily distributed across various subcellular compartments involved in cellular secretion and vesicle transport, including secretory granule membrane, secretory granule lumen, cytoplasmic vesicle lumen, and tertiary granules. Molecular function terms enriched for DEGs were mainly related to the involvement of these molecular functions in immune response and cellular metabolism, including immune receptor activity, complement receptor activity, and cytokine receptor activity (Figure 2; Table 2). KEGG analysis showed enrichment of DEGs in diverse biological activities such as NOD-like receptor signaling pathway, TNF signaling pathway *Staphylococcus aureus* infection, and Neutrophil extracellular trap formation (Figure 3; Table 3).

**Table 2:** Results from GO enrichment analysis of DEGs.

ID	Ontology	Description	Adjust *p*-value	GeneID	Count
GO:0001819	BP	Positive regulation of cytokine production	0.002	IL1R1/HMGB2/IL17RA/NOD2/CASP1/NAIP/CD58/AIM2/C5AR1/HPSE/IL18R1/LILRA2/IL6R/CD2/RGCC/CD3E	16
GO:0002430	BP	Complement receptor mediated signaling pathway	0.003	FPR1/FPR2/CR1/C5AR1	4
GO:0042742	BP	Defense response to bacterium	0.005	CAMP/HMGB2/LTF/FPR2/NOD2/NAIP/ANXA3/LCN2/C5AR1/GBP2/PRKCD/IL6R/DEFA4	13
GO:0002253	BP	Activation of immune response	0.005	FPR1/FPR2/CR1/NOD2/AIM2/C5AR1/MICB/PLSCR1/PRKCD/LILRA2/C1RL/RGCC/CD3E	13
GO:0032103	BP	Positive regulation of response to external stimulus	0.005	HMGB2/FPR2/IL17RA/NOD2/CASP1/NAIP/AIM2/C5AR1/PLSCR1/LILRA2/DDX60/IL6R/NMI/SNCA	14
GO:0050727	BP	Regulation of inflammatory response	0.005	MMP9/IL1R1/FPR2/IL17RA/NOD2/CASP1/NAIP/AIM2/PRKCD/BST1/BCL6/NMI/SNCA	13
GO:0002237	BP	Response to molecule of bacterial origin	0.005	CAMP/HMGB2/TRIB1/LTF/NOD2/CASP1/C5AR1/FOS/CHMP5/LILRA2/DEFA4/SNCA	12
GO:0002831	BP	Regulation of response to biotic stimulus	0.005	HMGB2/TRIB1/LTF/FPR2/CR1/NOD2/AIM2/MICB/PLSCR1/LILRA2/DDX60/NMI	12
GO:0002251	BP	Organ or tissue specific immune response	0.005	CAMP/LTF/NOD2/IL6R/DEFA4	5
GO:0031349	BP	Positive regulation of defense response	0.005	HMGB2/FPR2/IL17RA/NOD2/CASP1/NAIP/AIM2/PLSCR1/LILRA2/NMI/SNCA	11
GO:0019221	BP	Cytokine-mediated signaling pathway	0.005	IL1R1/IL17RA/CASP1/NAIP/SP100/AIM2/IL10RB/TANK/IL18R1/LILRA2/IL6R/NMI/ADIPOR1/IL2RB	14
GO:0071216	BP	Cellular response to biotic stimulus	0.005	CAMP/HMGB2/TRIB1/LTF/NOD2/CASP1/APAF1/CHMP5/LILRA2/DEFA4	10
GO:0007009	BP	Plasma membrane organization	0.007	MYOF/CR1/EPB41L3/MAFB/PLSCR1/PRKCD/CHMP5/MTSS1	8
GO:0071222	BP	Cellular response to lipopolysaccharide	0.008	CAMP/HMGB2/TRIB1/LTF/NOD2/CASP1/CHMP5/LILRA2/DEFA4	9
GO:0032496	BP	Response to lipopolysaccharide	0.008	CAMP/HMGB2/TRIB1/LTF/NOD2/CASP1/FOS/CHMP5/LILRA2/DEFA4/SNCA	11
GO:0030099	BP	Myeloid cell differentiation	0.008	MMP9/HMGB2/TRIB1/LTF/FOS/MAFB/JUNB/ALAS1/ACTN1/BCL6/KLF1/EPB42	12
GO:0002227	BP	Innate immune response in mucosa	0.008	CAMP/LTF/NOD2/DEFA4	4
GO:0071219	BP	Cellular response to molecule of bacterial origin	0.009	CAMP/HMGB2/TRIB1/LTF/NOD2/CASP1/CHMP5/LILRA2/DEFA4	9
GO:2000116	BP	Regulation of cysteine-type endopeptidase activity	0.009	MMP9/LTF/CFLAR/CASP1/NAIP/APAF1/AIM2/FIS1/SNCA	9
GO:0002274	BP	Myeloid leukocyte activation	0.009	CAMP/ANXA3/C5AR1/PLSCR1/PRKCD/LILRA2/NMI/CD2/SNCA	9
GO:0002221	BP	Pattern recognition receptor signaling pathway	0.009	LTF/NOD2/CASP1/NAIP/AIM2/LILRA2/DDX60/NMI	8
GO:0002833	BP	Positive regulation of response to biotic stimulus	0.009	HMGB2/FPR2/NOD2/AIM2/PLSCR1/LILRA2/DDX60/NMI	8
GO:0097529	BP	Myeloid leukocyte migration	0.009	IL1R1/FPR2/IL17RA/NOD2/DUSP1/C5AR1/BST1/IL6R/BSG	9
GO:0035456	BP	Response to interferon-beta	0.009	AIM2/XAF1/PLSCR1/CDC34	4
GO:0042581	CC	Specific granule	<0.001	CKAP4/CAMP/LTF/FPR2/ANXA3/LCN2/CEACAM8/HPSE/BST1/DEFA4	10
GO:0030667	CC	Secretory granule membrane	<0.001	CKAP4/FPR1/MGAM/FPR2/CR1/MME/CD58/C5AR1/ANPEP/CEACAM8/BST1/SNCA/BSG	13
GO:0070820	CC	Tertiary granule	<0.001	MMP9/CAMP/LTF/FPR1/MGAM/FPR2/CR1/CD58/CEACAM8	9
GO:0034774	CC	Secretory granule lumen	0.001	CAMP/LTF/APAF1/LCN2/HPSE/PRKCD/ACTN1/DEFA4/F13A1/APRT/PRDX6	11
GO:0060205	CC	Cytoplasmic vesicle lumen	0.001	CAMP/LTF/APAF1/LCN2/HPSE/PRKCD/ACTN1/DEFA4/F13A1/APRT/PRDX6	11
GO:0031983	CC	Vesicle lumen	0.001	CAMP/LTF/APAF1/LCN2/HPSE/PRKCD/ACTN1/DEFA4/F13A1/APRT/PRDX6	11
GO:0101003	CC	Ficolin-1-rich granule membrane	0.003	FPR1/MGAM/FPR2/CR1/CD58	5
GO:0035580	CC	Specific granule lumen	0.003	CAMP/LTF/LCN2/HPSE/DEFA4	5
GO:0061702	CC	Inflammasome complex	0.009	CASP1/NAIP/AIM2	3
GO:0140375	MF	Immune receptor activity	<0.001	IL1R1/FPR1/FPR2/IL17RA/CR1/C5AR1/IL10RB/IL18R1/LILRA2/IL6R/IL2RB	11
GO:0004875	MF	Complement receptor activity	0.005	FPR1/FPR2/CR1/C5AR1	4

DEGs, Differentially expressed genes; GO, Gene Ontology; BP, Biological processes; CC, Cellular components; MF, Molecular functions.

**Table 3:** Results from KEGG enrichment analysis of DEGs.

Description	Gene Ratio	Adjust *p*-value	Gene ID	Count
NOD-like receptor signaling pathway	10/84	<0.001	CAMP/NOD2/CASP1/NAIP/AIM2/GBP2/TANK/PRKCD/TXN/DEFA4	10
TNF signaling pathway	8/84	<0.001	MMP9/CFLAR/NOD2/PIK3CB/CREB5/FOS/IL18R1/JUNB	8
Hematopoietic cell lineage	7/84	<0.001	IL1R1/CR1/MME/ANPEP/IL6R/CD2/CD3E	7
*Staphylococcus aureus* infection	6/84	<0.001	KRT23/CAMP/FPR1/FPR2/C5AR1/DEFA4	6
Legionellosis	4/84	0.002	CR1/CASP1/NAIP/APAF1	4
Estrogen signaling pathway	6/84	0.002	MMP9/KRT23/PIK3CB/CREB5/FOS/PRKCD	6
Fluid shear stress and atherosclerosis	6/84	0.003	MMP9/IL1R1/DUSP1/PIK3CB/FOS/TXN	6
Neutrophil extracellular trap formation	7/84	0.003	CAMP/FPR1/FPR2/CR1/PIK3CB/CASP1/C5AR1	7
Th17 cell differentiation	5/84	0.004	IL1R1/FOS/IL6R/IL2RB/CD3E	5
Lipid and atherosclerosis	7/84	0.005	MMP9/ABCG1/PIK3CB/CASP1/APAF1/FOS/TANK	7
Human T-cell leukemia virus 1 infection	7/84	0.006	IL1R1/ETS2/PIK3CB/CREB5/FOS/IL2RB/CD3E	7
Coronavirus disease - COVID-19	7/84	0.008	PIK3CB/CASP1/C5AR1/FOS/IL6R/F13A1/RPS28	7
Osteoclast differentiation	5/84	0.009	IL1R1/PIK3CB/FOS/JUNB/LILRA2	5

DEGs, Differentially expressed genes; KEGG, Kyoto Encyclopedia of Genes and Genomes; COVID-19, Coronavirus disease 2019.

### 3.3 PPI network establishment and identification of candidate hub genes

**Figure 4:**
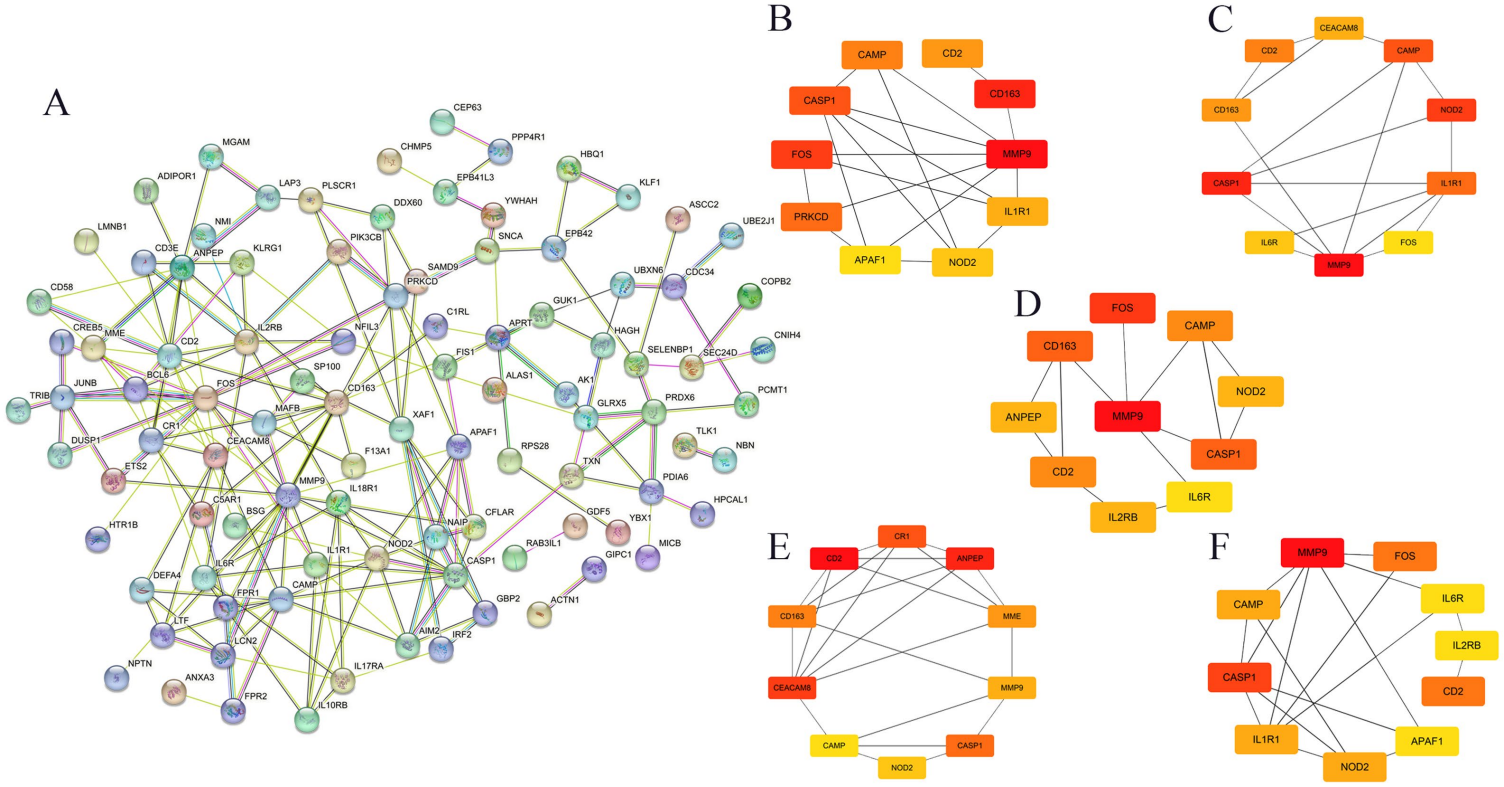
The PPI network and cluster analysis of co-expressed DEGs in GBS and COVID-19. **(A)** Functional protein–protein association networks of co-expressed DEGs in GBS and COVID-19 analysed and selected by STRING online database. **(B–F)** The top 10 hub genes were identified according to five different algorithms (Closeness, EPC, Degree, MCC, and MNC, respectively) using cytoHubba, which are crucial for understanding the central elements within the biological networks of both diseases.

Given the importance of immune response and inflammation in GBS and COVID-19, we employed ssGSEA to evaluate immune cell abundance in healthy individuals and patients. After eliminating DEGs with weak interactions, 96 co-expressed DEGs were retained (Figure 4A). Furthermore, the CytoHubba plugin in Cytoscape (version 3.9.1) was employed, utilizing five different algorithms (MNC, MCC, EPC, Degree, and Closeness) to identify the top 10 hub DEGs (Figures 4B–F). Ultimately, 3 co-expressed DEGs were selected as NETRGs (MMP9, CAMP, and CASP1) for further analysis, as they were shared among the different algorithms and NETs-related genes.

### 3.4 Analysis of immune infiltrating cells and correlation with hub genes

**Figure 5:**
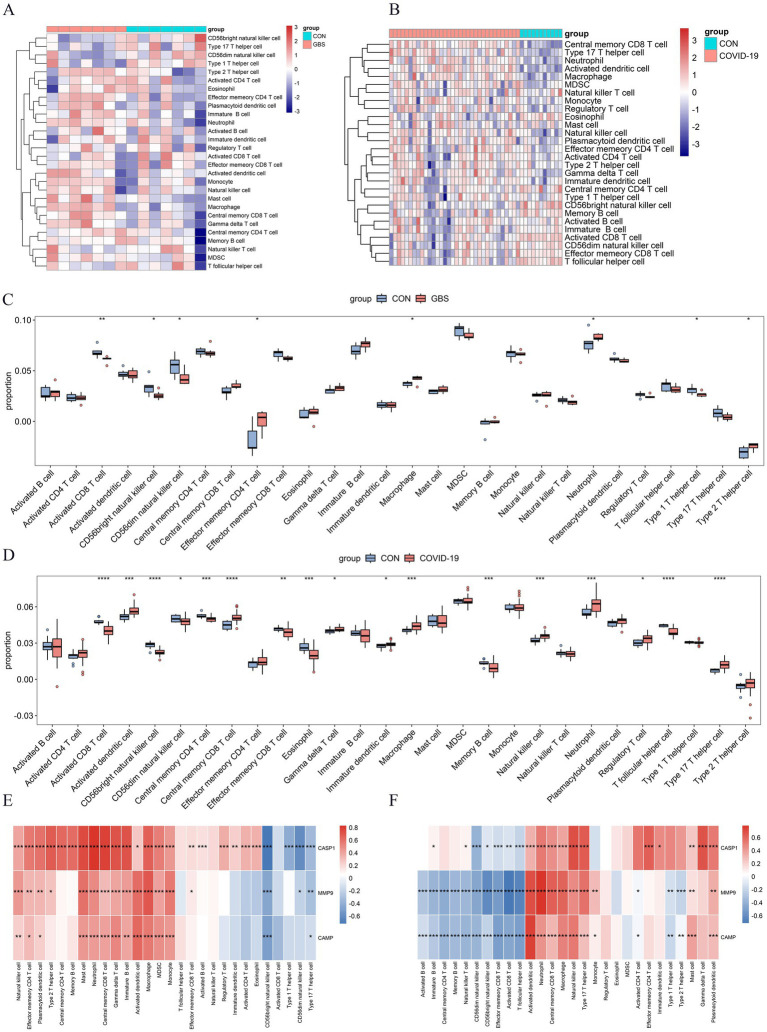
Analysis of Immune cell infiltration in GBS and COVID-19 datasets, and their correlation with NETRGs. **(A,B)** Heat map of the Immune cell infiltration analysis in GSE31014 and GSE213313 datasets: blue indicates a relatively low expression, and red indicates a relatively high expression. **(C,D)** Immune cell infiltration analysis in GSE31014 and GSE213313 datasets comparing patients and control samples. The correlation analysis reveals significant associations between specific immune cells (e.g., macrophages, neutrophils) and the identified NETRGs, highlighting the role of these immune cells in the pathogenesis of both diseases. **(E,F)** Correlation analysis between NETRGs and infiltrating immune cells. * indicates *p* < 0.05, ** represents *p* < 0.01,*** represents *p* < 0.001, blank represents no significant difference. Blue indicates a relatively negative correlation, and red indicates a relatively positive correlation.

Based on functional enrichment, hub gene identification, and existing literature, immune response and inflammation are crucial in GBS and COVID-19 development. To explore the intricate immune landscape of these diseases, we used ssGSEA to assess immune cell abundance in healthy individuals and patients (Figures 5A,B). In the context of the GBS-related dataset, positive correlations were observed with effector memory CD4 T cells, macrophages, neutrophils, and type 2 T helper cells. Conversely, negative correlations were evident with activated CD8 T cells, CD56bright natural killer cells, CD56dim natural killer cells, and Type 1 T helper cells (Figure 5C). For COVID-19 patients (Figure 5D), predominantly positive immune cell expression was observed, including activated dendritic cells, central memory CD8 T cells, gamma delta T cells, immature dendritic cells, macrophages, natural killer cells, neutrophils, regulatory T cells, and type 17 T helper cells. In addition to negative associations found in the GBS-related dataset, COVID-19 infections also displayed negative correlations with central memory CD4 T cells, effector memory CD8 T cells, eosinophils, memory B cells, and T follicular helper cells (Figure 5D). Furthermore, Figures 5E,F illuminated the correlations between the NETRGs and immune cell infiltrations. In both GBS and COVID-19 datasets, the correlation analysis demonstrated a positive association linking macrophage, neutrophil, and central memory CD8 T cell activity with NETRGs. Conversely, a negative correlation was observed between CD56bright natural killer cells and the NETRGs across both diseases.

### 3.5 Validation of the NETRGs

**Figure 6:**
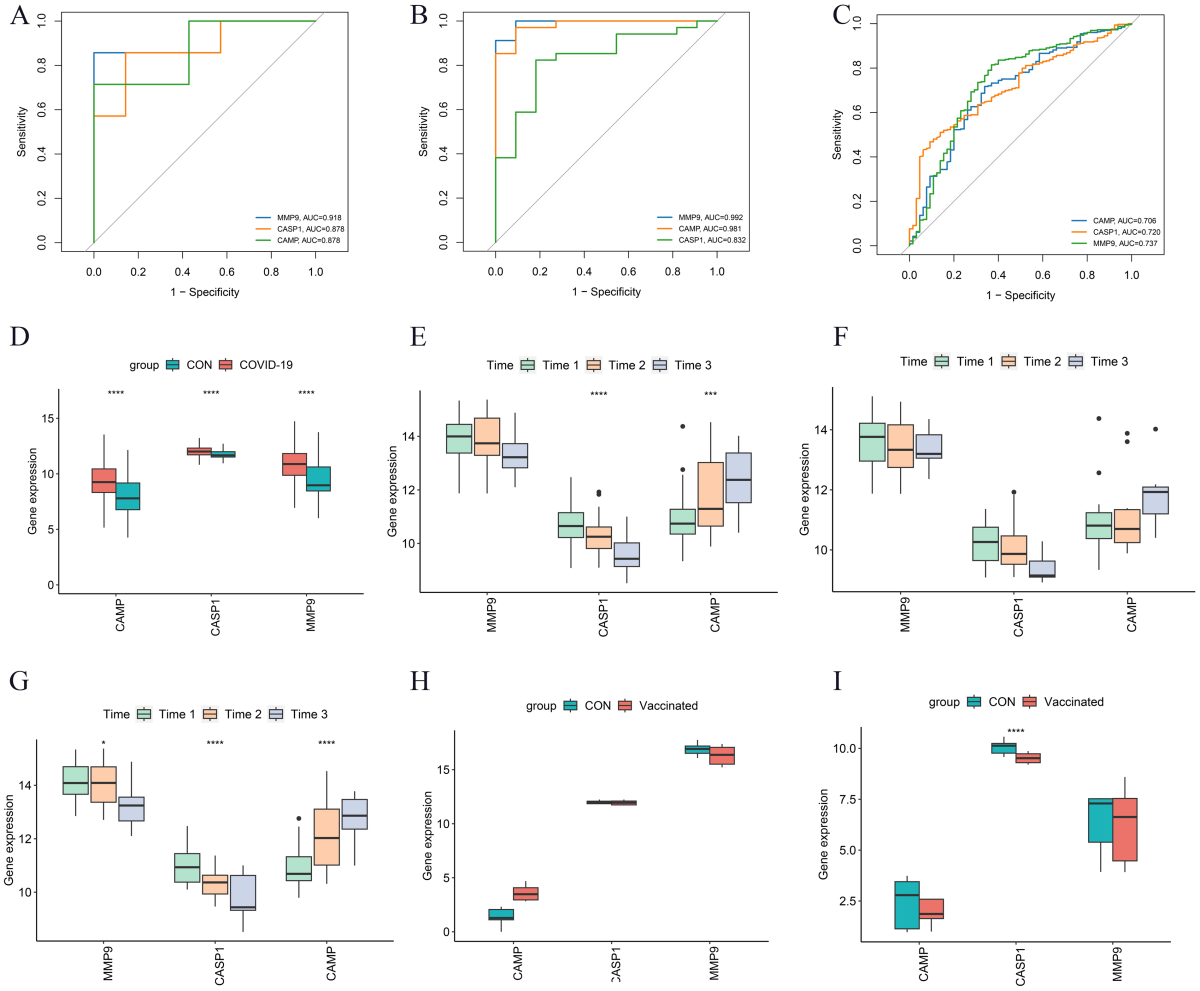
Further analysis and validation of the NETRGs. **(A–C)** ROC curves of the NETRGs expression levels discriminating specific patients (GBS and COVID-19) and control individuals in GSE31014 datasets, GSE213313 datasets, and GSE215865 datasets. The ROC curves demonstrate the diagnostic performance of these genes in distinguishing GBS and COVID-19 patients from controls, with MMP9 showing particularly high diagnostic accuracy. **(D)** The NETRGs expression levels in GSE215865 datasets comparing COVID-19 patients and control samples shown by boxplot. **(E–G)** The expression levels of NETRGs over time in GSE213313 dataset for all COVID-19 patients, non-critical COVID-19 patients, and critical COVID-19 patients. Time1 to Time3 correspond to increasing disease duration. **(H,I)** The NETRGs expression levels in GSE191088 and GSE200274 datasets comparing individuals with SARS-CoV-2 vaccine and control samples shown by boxplot. The boxplots illustrate the expression patterns of NETRGs across different datasets and patient groups, revealing temporal dynamics and differences in expression levels related to disease progression **(D–G)** and vaccination status **(H,I)**.

The NETRGs exhibited excellent diagnostic performance within both GBS and COVID-19 discovery sets, as evidenced by the high AUC values (Figures 6A,B). Particularly, MMP9 showed the highest diagnostic performance in both diseases, with AUC values of 0.918 and 0.992 for GBS and COVID-19, respectively. The diagnostic potential of the NETRGs persisted in the validation dataset GSE215865. Here, the ROC curve illuminated the ability of NETRGs to effectively diagnose COVID-19 patients, with MMP9 once again leading the pack, displaying a noteworthy AUC of 0.737 (Figure 6C). Furthermore, the expression patterns of the NETRGs within dataset GSE215865 were consistent with the discovery datasets, demonstrating consistent upregulation of all NETRGs in COVID-19 patients (Figure 6D). Within the discovery set, the boxplot analysis of NETRG expressions revealed a temporal decline in the expression levels of MMP9 and CASP1 (*p* > 0.05 and *p* < 0.05, respectively), while the expression level of CAMP exhibited an increase (Figure 6E). Notably, a similar temporal expression pattern of NETRGs was significantly observed among critical patients in discovery set (Figures 6F,G). In contrast, the levels of MMP9, CASP1, and CAMP expression exhibited a gradual decline over time (*p* < 0.05) within the validation dataset GSE195796 (Figure 6H). Datasets GSE191088 and GSE200274, which focused on inactivated and mRNA vaccination for COVID-19, were obtained to effectively determine the involvement of the NETRGs in the progression of COVID-19 vaccines. Among the analyzed genes, CASP1 emerged as the sole gene displaying a significant difference in expression levels between vaccinated and unvaccinated individuals. Notably, this difference was in contrast to the observations made within the discovery set (Figures 6I,J).

### 3.6 Small molecule drug candidates for comorbidity prevention

Using the previously mentioned PPI network, we extracted 96 co-expressed DEGs and mapped them to the cMap database to identify potential therapeutic compounds for COVID-19-related GBS (Table 4). The top 10 small-molecule compounds, BI-2536, TG-101348, and XMD-885, were synthesized in this study and exhibited promising scores. BI-2536 obtained the highest score (−97.4) and TG-101348 ranked second with a close score of (−96.9).

**Table 4:** Top ten drug candidates selected by cMAP.

Rank	Score	Name	Description
1	−97.4	BI-2536	PLK inhibitor
2	−96.9	TG-101348	FLT3 inhibitor
3	−95.96	XMD-885	Leucine rich repeat kinase inhibitor
4	−93.76	AZD-8055	MTOR inhibitor
5	−93.25	VER-155008	HSP inhibitor
6	−91.16	Montelukast	Leukotriene receptor antagonist
7	−90.66	PIK-75	DNA protein kinase inhibitor
8	−90.25	Fostamatinib	SYK inhibitor
9	−90.09	XMD-892	MAP kinase inhibitor
10	−89.97	ISOX	HDAC inhibitor

## 4 Discussion

The genetic susceptibility of GBS in the context of COVID-19 infection remains poorly understood, necessitating comprehensive research efforts. In response to this knowledge gap, our study aimed to employ bioinformatics analysis to elucidate potential co-expressed genes, regulatory targets, pathways, and therapeutic molecules shared between GBS and COVID-19. Through rigorous analysis, 145 co-expressed DEGs between COVID-19 and GBS were identified, and functional enrichment analysis demonstrated that inflammatory pathways and immune activation were involved in the progression of the two diseases. Afterward, we identified three key co-expressed hub genes that are associated with NETs, which may offer potential avenues for the development of effective therapeutic management strategies for post-COVID-19 GBS.

The lack of data detecting the virus in cerebrospinal fluid supports an immune mechanism rather than direct infection (26). It is suggested that post-COVID-19 GBS could be caused by an immune response triggered by similarities between SARS-CoV-2 and human gangliosides, resulting in a cross-reaction that leads to immune-mediated nerve damage and subsequent development of GBS (7). “Cytokine storm” released by SARS-CoV-2 is another proposed mechanism of the autoimmune pathway that possibly could result in post-COVID-19 GBS (15). In our analysis, the GO analyses on co-expressed DEGs in COVID-19 and GBS, indicating enrichment in immune response processes, cellular components related to protein secretion and signaling, and molecular functions associated with immune and cytokine production activities. KEGG pathways suggest a strong involvement of immunity and inflammation in the pathogenesis of both COVID-19 and GBS, further emphasizing the centrality of immunity and inflammation in post-COVID-19 GBS pathogenesis. It is noteworthy that autoimmunity and hyper-inflammation are linked to acute SARS-CoV-2 infection, while an insufficient immune response or dysfunction may contribute to long COVID (27). Given that studies suggest GBS is an autoimmune disease, it is crucial to recognize the significance of addressing post-COVID-19 GBS in the realm of long-term complications, especially considering the prolonged period of immune dysregulation following SARS-CoV-2 infection.

Neutrophils recruited as one of the earliest immune cells during microbial infection and inflammation, perform crucial immune functions beyond pathogen cytotoxicity (28). The NET formation, a process called NETosis, has been proven to play a detrimental role in the pathophysiology of many infectious and noninfectious diseases. A significant prerequisite for NETosis is the mobilization of neutrophils from the bone marrow to the site of inflammation, which is indicated by a high proportion of neutrophils in the blood. Macrophages, crucial effectors of the innate immune response against infection and inflammation, are recruited to the loci of NETosis, where they swallow apoptotic neutrophils. We found that macrophages and neutrophils had more significant proportions among immunological categories in the COVID-19 and GBS datasets, indicating their crucial roles in early inflammatory responses and antiviral defense. Another study also showed that the dysregulated myeloid responses to SARS-CoV-2 infection in severe COVID-19 patients had increased levels of neutrophils and macrophages (29). Similarly, higher neutrophil ratios and counts were significantly associated with acute-onset GBS, recurrent GBS patients, and disease severity (30). Notably, it has been discovered that the presence of macrophages promotes the development of the disease in an experimental autoimmune neuritis (EAN) model. Macrophages participate in the demyelination process in EAN/GBS by releasing proinflammatory cytokines such as TNF-*α*, IL-12, and IL-6 (31). Considering the involvement of the NETs pathway, which was a potential shared pathogenic pathway in GBS and COVID-19, we hypothesized that the infiltration of neutrophils and macrophages in peripheral blood may serve as key immune cells during early inflammatory responses and virus resistance. Additionally, it may be associated with NETosis against infections and inflammation in post-COVID-19 GBS patients. Therefore, these findings suggested shared altered immune cell responses and suggested NETs may mainly drive disease progression in post-COVID-19 GBS.

In our study, lower expression of active CD8 T cells, CD56bright natural killer cells, and CD56dim natural killer cells were observed in both COVID-19 infection and GBS patients. The delayed and targeted T-cell response is essential in mounting an effective immune defense against viral infections and inflammation. The active CD8 T cells in particular are known to produce a delayed and focused T cell response, acting as cytotoxic effector cells. The reduced percentage of COVID-19 and GBS patients during the acute phase may be explained by this. However, CD56 bright natural killer cells and CD56dim natural killer cells were found depleted in all COVID-19 samples in blood samples (32), which may be mainly recruited in early immune and adaptive responses. Interestingly, CD56+ natural killer cells were found to decrease in the peripheral blood of individuals with inflammation and autoimmune disorders, but higher frequency of these cells in organs such as the liver, gut, and lung (33). The decrease in the percentage of CD56+ natural killer cells in peripheral blood also suggested that these cells may be involved in systemic autoimmune and localized specific inflammation.

As a result, the identification of three NETRGs (MMP9, CASP1, and CAMP) showcased relatively optimal diagnostic biomarkers were identified based on the PPI networks, as the ROC curves showed. MMP9, a member of the zinc-dependent endopeptidase family, is involved in the degradation of extracellular matrix (ECM) molecules and plays a role in the activation of the immune system and regulation of the inflammatory cascade in the development of different disorders (34, 35). The activity of MMP9 promotes the progression of NETs, such as angiogenesis, through the direct release of growth factors and cleavage of ECM molecules, making MMP9 a promising targeted therapy for NETs. There have been reported that MMP9 is not only associated with the severity and mortality of COVID-19 but also with venous thromboembolism, chronic myocardial fibrosis, and susceptibility to COVID-19-related neurologic syndrome (36–40). By interacting with laminin in the ECM, the dystroglycan (DG) complex in the context of peripheral nerves plays a vital role in myelin development and stability. However, the upregulation of MMP9 in the EAN model induces autoimmune neuritis by cleaving *β*-DG (a component of the DG complex), which elucidates the involvement of MMP9 in mediating inflammatory demyelination and cellular infiltration in GBS (41). Perhaps, the changes in MMP9 may not only explain the involvement in post-COVID-19 GBS but may allow for the identification and novel alternate therapeutic approach against this disease.

NETs not only play a crucial role in host defense, but NETosis was also widely detected in various organ tissues and inflammatory diseases, where they significantly contribute to disease pathology. NETosis, a process dependent on the peptidyl arginine deiminase 4 (PAD4)-mediated post-translational modification of histones, is facilitated by the formation of the NOD-like receptor family, pyrin domain containing 3 (NLRP3) inflammasome in neutrophils. Notably, even in the absence of infection, blocking caspase-1 (CASP1), the effector molecule of the NLRP3 inflammasome, drastically lowers NETosis in human neutrophils (42). Active NLRP3 inflammasome was found in PBMCs and tissues of 124 SARS-CoV-2 patients, and its presence was associated with disease severity, suggesting it is a potential therapeutic target for COVID-19 (43). The upregulation of CASP1 increased in parallel with the levels of IL-18 in the EAN model, also the level of IL-18 in GBS patients was demonstrated significantly higher compared to controls in cerebrospinal fluid. Our finding further supported the notion of strong activation of CASP1 in both COVID-19 and GBS patients at the transcriptomic level.

Besides, we identified the CAMP gene as another member of the NETRGs. This gene encodes human cathelicidin (LL-37), an exceptional antimicrobial peptide that exhibits widespread expression in neutrophils, bone marrow, and nasal epithelium. LL-37 plays a regulatory role in immune responses by promoting immune cell migration, modulating cytokine release, and facilitating coordination between innate and adaptive immunity (44). Additionally, LL-37 encourages the development of NETs, and it has been suggested that disorders including cancer and autoimmune reactions are caused by the disruption of its expression. It is important to note that elevated levels of LL-37 may cause hypercoagulation in COVID-19 patients by activating coagulation factors (45), even though LL-37 has been reported to act as a preventive and therapeutic strategy for SARS-CoV-2 infections (46), by facilitating effective NETs clearance by macrophages and speeding endothelial repair after inflammatory tissue damage. The upregulation of CAMP in both COVID-19 and GBS patients was observed in our study, but it is not clear whether the positive immunomodulatory activity directly impacts disease outcomes or if the deregulation of expression leads to a negative effect. Considering the evident activation of the NETs pathway and other immune-related pathways found in discovery datasets, it is worth noting the potential hyperinflammation impact of CAMP on post-COVID-19 GBS. Further studies are needed to clarify the relationship between the upregulation of CAMP expression and immune response outcomes, as well as to elucidate the pathophysiological mechanism in post-COVID-19 GBS patients.

Given the temporal dependency in both COVID-19 and GBS, we investigated the relationship between NETRGs and temporal changes in COVID-19 patients. The expression levels of MMP9 and CASP1 displayed a temporal decline, whereas the expression level of CAMP exhibited an increase over time. Nevertheless, there was no significant correlation between CAMP expression and time in non-critical patients, while a correlation was observed in critical infections found in our study. We examined gene expression levels in an independent validation dataset to confirm these results, and the results showed that all three genes saw their expression levels decline with time. It’s possible that within 15 days, every patient in the discovery set was in the acute stage of the infection, with the proportion of patients who were very ill rising over time. As aforementioned, it is worth considering whether the increase in CAMP indicates a protective effect or immune imbalance in COVID-19 patients. Since the commencement of the vaccination campaign, a debate has emerged concerning a potential correlation between GBS, other neuropathies, and the SARS-CoV-2 vaccines. Despite the attention given to GBS cases associated with COVID-19 and its vaccines, the exact underlying mechanism connecting GBS with SARS-CoV-2 infection or vaccination remains elusive (7, 47). However, we discovered that only CASP1’s expression, which had been downregulated, differed significantly from that of unvaccinated people. It appears that the NETRGs discovered in our study may not trigger vaccine-induced GBS related to COVID-19.

To date, the prevailing course of action for managing post-COVID-19 GBS has been the administration of intravenous immunoglobulins (2), by the established treatment protocol for GBS. However, despite getting this standard treatment, there have been situations where individuals showed persistent neurological impairment or a static neurological condition (48). Given the presence of residual defects and poorer clinical outcomes, it is crucial to explore potential biological treatments that aim to modulate the immunopathology of post-COVID-19 GBS. Through PPI networks, we identified 96 commonly expressed genes that were used to predict potential drugs by screening the cMAP database. Our findings demonstrate the potential of these small-molecule compounds to target inflammatory responses and offer promising therapeutic strategies. However, it is important to acknowledge the limitations of our study. These include potential bias brought on by the use of database samples and computational biology technologies. To validate the three identified NETRGs, future research should incorporate a larger sample size that encompasses a broader spectrum of diseases. Furthermore, *in vivo,* studies are warranted to confirm the functional role of the transcriptional signatures and the candidate drugs, particularly in long COVID individuals.

## 5 Conclusion

In conclusion, our study provides valuable insights into the potential involvement of NETs formation in the pathogenesis of post-COVID-19 GBS. The identification of MMP9, CASP1, and CAMP as key genes associated with NETs highlights their relevance in the pathogenesis of GBS following COVID-19 infection. These findings underscore the significance of targeting NETs as a therapeutic approach in post-COVID-19 GBS. However, further investigations are needed to unravel the precise functional roles of these genes and to ascertain the clinical implications of modulating NETs formation in patients afflicted with post-COVID-19 GBS. Our findings contribute to a deeper comprehension of the intricate interaction between COVID-19 and GBS by illuminating these fundamental mechanisms, opening the door for new interventions that can enhance patient outcomes and direct future therapeutic approaches.

## Data Availability

The original contributions presented in the study are included in the article/supplementary material, further inquiries can be directed to the corresponding author.

## Footnotes

^1^ http://www.ncbi.nlm.nih.gov/gds/

^2^ https://clue.io/
